# Interobserver analysis of safety practices and behaviors adopted by elderly people to prevent falls

**DOI:** 10.1590/1518-8345.3209.3268

**Published:** 2020-06-01

**Authors:** Cristina Rosa Soares Lavareda Baixinho, Maria dos Anjos Coelho Rodrigues Dixe, Carla Madeira, Silvia Alves, Maria Adriana Henriques

**Affiliations:** 1 Escola Superior de Enfermagem de Lisboa, Fundamentos de Enfermagem, Lisboa, Lx, Portugal.; 2 Instituto Politécnico de Leiria, Escola Superior de Saúde, Leiria, Lr, Portugal.; 3 Hospital de VIla Franca de Xira, Medicina, Vila Franca de Xira, VFX, Portugal.; 4 Hospital de VIla Franca de Xira, Unidade de Cuidados Intensivos, Vila Franca de Xira, VFX, Portugal.; 5 Escola Superior de Enfermagem de Lisboa, Enfermagem Comunitária, Lisboa, LX, Portugal.

**Keywords:** Geriatric Nursing, Validation Studies, Psychometrics, Accidental Falls, Elderly, Home for the Aged, Enfermagem Geriátrica, Estudos de Validação, Psicometria, Acidentes por Quedas, Idosos, Instituição de Longa Permanência para Idosos, Enfermaría Geriátrica, Estudios de Validación, Psicometria, Accidentes por Caídas, Ancianos, Hogares para Ancianos

## Abstract

**Objective:**

determine the psychometric properties of the safety practices and behaviors dimension of the Scale of Practices and Behaviors of Institutionalized Elderly People to Prevent Falls in a sample of elderly people with cognitive decline.

**Method:**

methodological study, with a quantitative approach, to assess the psychometric properties of the mentioned scale in a sample with 102 elderly people with cognitive decline who lived in two long-term care institutions for the public in this age group. Internal consistency evaluation was carried out by calculating the Cronbach’s alpha coefficient; interobserver reliability was expressed by Cohen’s kappa coefficient; and temporal stability, by obtaining Spearman correlation. Compliance with all ethical procedures was observed.

**Results:**

the dimension of safety practices and behaviors showed α = 0.895 for its 11 items. Seven out of the 11 items reached good to excellent agreement among the experts for interobserver reliability. Kappa index values indicated that the instrument is valid and reliable. Safety practices and behaviors were influenced by institutionalization time, being at least 85 years old, and gait skills.

**Conclusion:**

the results pointed out that the instrument has good reproducibility and is valid and reliable, which allows its use in clinical practice in elderly people with cognitive decline as well as in research.

## Abstract (pt)

**Objetivo:**

determinar as propriedades psicométricas da dimensão das práticas e dos comportamentos de segurança da Escala de Práticas e Comportamentos dos Idosos Institucionalizados para Prevenir Quedas numa amostra de idosos com declínio cognitivo.

**Método:**

estudo metodológico, com abordagem quantitativa, para a avaliação das propriedades psicométricas da escala numa amostra de 102 idosos com declínio cognitivo, residentes em duas Instituições de Longa Permanência para Idosos. A avaliação da consistência interna foi efetuada pelo α de Cronbach, a confiabilidade interobservadores, calculada pelo coeficiente de concordância *Kappa* de Cohen e a estabilidade temporal, pela correlação de Spearman. Todos os procedimentos éticos foram contemplados no estudo.

**Resultados:**

a dimensão das práticas e dos comportamentos de segurança da escala apresentou um α=0,895 para os seus 11 itens. Na confiabilidade interobservador, 7 dos 11 itens obtiveram de boa a excelente concordância entre os juízes. Os valores do Índice *Kappa* evidenciam que o instrumento é válido e fidedigno. As práticas e os comportamentos de segurança são influenciados pelo tempo de institucionalização, idade superior a 85 anos e capacidade de marcha.

**Conclusão:**

os resultados indicam uma boa reprodutibilidade, o instrumento é válido e fidedigno, permitindo a utilização do instrumento na clínica em idosos com declínio cognitivo e na investigação.

## Abstract (es)

**Objetivo:**

determinar las propiedades psicométricas de la dimensión de prácticas y conductas de seguridad de la Escala de prácticas y comportamientos de los ancianos institucionalizados para prevenir las caídas, sobre una muestra de ancianos con deterioro cognitivo.

**Método:**

estudio metodológico de abordaje cuantitativo para evaluar las propiedades psicométricas de la escala sobre muestra de 102 ancianos con deterioro cognitivo, residentes en dos hogares para ancianos. Evaluación de consistencia interna verificada por α de Cronbach, confiabilidad interobservadores calculada por coeficiente de concordancia Kappa de Cohen, estabilidad temporal comprobada por correlación de Spearman. Fueron contemplados todos los procedimientos éticos.

**Resultados:**

la dimensión de prácticas y comportamientos de seguridad de la escala expresa α=0,895 para sus 11 ítems. En confiabilidad interobservadores, 7 de los 11 ítems obtienen buena o excelente concordancia entre los evaluadores. Los valores del índice Kappa evidencian que el instrumento es válido y fidedigno. Las prácticas de comportamiento de seguridad están influidas por el tiempo de institucionalización, edad superior a 85 años y capacidad de marcha.

**Conclusión:**

los resultados demuestran buena reproductividad, el instrumento es válido y confiable, pudiéndoselo utilizar en la clínica con ancianos con deterioro cognitivo y en la investigación.

## Introduction

Falls, being the main accident in the elderly population, are considered a serious public health issue. Because of their consequences in functioning and the dependence and loss in quality of life they cause^(^ 1 ^)^, they are one of the main causes of death, disability, and costs to the health system, accounting for 85% of traumas in elderly people^(^ 2 ^)^.

This problem is difficult to control because of the multifactorial nature of its genesis, which imposes the need for prevention. Despite this difficulty, researchers have emphasized that most risk factors are modifiable by introducing single or multiple interventions oriented toward one or more risk factors^(^ 1 ^-^ 6 ^)^.

One example is the environmental changes in elderly people’s houses. It is known that nearly every house has fall risk factors for elderly people because of deficient lighting and the presence of rugs and obstacles that reduce indoor accessibility and mobility, among other problems^(^ 3 ^)^. Environmental risks can be minimized or eliminated by implementing behavioral changes in the elderly population in combination with modifications in the environment, resulting in a positive impact on the reduction of risks and on the prevalence of falls in elderly people’s houses^(^ 4 ^-^ 5 ^)^.

When mentioning the difficulties faced to carry out preventive actions, some authors have criticized the role played by research. They have noticed that the biomedical model has controlled evidence production in the fall research field, imposing the positivist research paradigm^(^ 6 ^-^ 7 ^)^.

This approach has offered gains, but researchers have stressed that it does not cover the complexity of fall events, their communication and the knowledge regarding elderly people’s practices and behaviors concerning their communication, safe self-care practices, and other behavioral factors that may increase the chances of falls to occur^(^ 6 ^-^ 7 ^)^.

Consequently, it is necessary to explore other dimensions of the phenomenon and new interventions^(^ 6 ^)^. This is a question that pertains not only elderly people who live in the community, but mostly those who are institutionalized, given that they are more vulnerable, less independent, and more frequently affected by chronic diseases^(^ 1 ^,^ 6 ^)^. Most institutionalized elderly people have two or more comorbidities, with high blood pressure (55.8%), dementia syndrome (18.3%), diabetes mellitus (16.3%), and Alzheimer’s disease (14.4%) being the most common diagnoses^(^ 1 ^)^. Polymedication adds to the list of risk factors, with 52% of the residents of long-term care institutions (LTCIs) taking eight or more different medications a day^(^ 1 ^)^.

Institutionalized elderly people also have to deal with the change in environment and the presence of employees and other elderly people, which are risk factors specific to LTCIs^(^ 1 ^)^.

Additionally, more dependent, institutionalized elderly people have worse prevention practices and behaviors^(^ 6 ^)^, especially regarding self-care. Elderly people showing functional disability in one to five activities of daily living had a probability to fall 46% higher, and those showing functional disability in all activities of daily living had lower chances to fall (prevalence ratio = 0.57; 95% confidence interval: 0.34 - 0.96)^(^ 8 ^)^. Fall risk increases progressively with the increase in the level of dependence, except in totally dependent elderly people^(^ 9 ^)^.

It is important to emphasize that cognitive alterations and deficits lead to functional decline, with a reduction and/or loss of skills, significantly interfering with practices and behaviors, which increases fall risk directly or indirectly.

Finding out the practices and behaviors of people with cognitive decline contributes to a more comprehensive discussion on fall risk dimensions other than biophysiological ones and may bring new elements to the debate about fall prevention measures in this public. The authors of the present study did not find instruments validated to examine practices and behaviors of institutionalized elderly people with cognitive decline to prevent falls in the literature.

The main objective of the present study was to determine the psychometric properties of the safety practices and behaviors dimension of the Scale of Practices and Behaviors of Institutionalized Elderly People to Prevent Falls (SPBIEPPF) in a sample of elderly people with cognitive decline.

## Method

Methodological study, with a quantitative approach, to assess the psychometric properties of the SPBIEPPF safety practices and behaviors dimension in a sample of elderly people with cognitive decline.

The SPBIEPPF has two dimensions: the first concerns to practices and behaviors of bilateral communication between elderly people and different professionals at LTCIs, and the second is related to safety practices and behaviors adopted by elderly people. The latter dimension is made up of two factors, one pertaining safety practices and behaviors in self-care (first seven items) and another addressing practices and behaviors related to the accessibility in the physical space (last four items). Each item scores from 1 (never) to 5 (always). This scale was designed by Portuguese researchers and validated in a sample with elderly people without cognitive decline who lived in six Portuguese LTCIs^(^ 10 ^)^.

In the validation study of the scale, in which the elderly people themselves filled out the instrument^(^ 10 ^)^, both dimensions showed good internal consistency: the six items in the communication subscale obtained α = 0.881, and in the adopted safety practices and behaviors dimension the 11 items provided α = 0.817^(^ 10 ^)^.

The SPBIEPPF communication practices and behaviors dimension scores between 6 and 30 points, and the safety practices and behaviors adopted by elderly people dimension from 11 to 55 points^(^ 10 ^)^.

The psychometric properties of the scale showed that it was reliable and measured the examined variables properly^(^ 10 ^)^ for the sample of conscious and instructed elderly people with which it was validated. These properties were determined for each scale dimension^(^ 10 ^)^ to allow its use in modules, according to the evaluation needs.

The importance of assessing safety practices and behaviors in populations with cognitive decline justifies the validation of this dimension by observation.

For the present study, the number of answer options was changed from five to three (never, sometimes, and always) to make its application easier with the observation method, a need imposed by the presence of cognitive decline in the target population.

The population of the present study was 204 elderly people who lived in two LTCIs, which authorized its execution. The final sample was 102 elderly people who met the predefined inclusion criteria: being at least 65 years old, being institutionalized, and showing cognitive decline as assessed by applying the Mini-Mental State Examination (MMSE, version in Portuguese)^(^ 11 ^)^: ≤ 15 points for illiterate people, ≤ 22 for people with up to 11 years of formal education, and ≤ 27 for people with more than 11 years of formal education)^(^ 11 ^)^.

Elderly people who were totally dependent to carry out activities of daily living and those who experienced falls during the study period because of the influence of the fear of falling on the residents’ practices and behaviors were excluded. The level of dependence was evaluated by obtaining the Barthel index by applying the instrument validated for the Portuguese population^(^ 12 ^)^. The elderly people who could walk around were evaluated regarding the use or not of an auxiliary gait device.

Data collection occurred between March and November 2017 and was carried out by four nurses specialized in rehabilitation nursing, two at each institution. First, a training addressing the scale structure, its variables, and the observation application mode was offered to the nurses. Subsequently, a pretest was performed, with the application of the scale to seven elderly people with cognitive decline who were not included in the final sample to train the professionals and make it possible to identify difficulties in the observation process and the completion of the instrument.

Last, the scale was applied by the professionals, who were independent observers, with a one-week interval between the evaluation of each one of them. Consequently, for interobserver reliability analysis, each elderly person was watched by two professionals while carrying out activities of daily living, over one week, in different moments. Fall occurrence was monitored and registered in the clinical process.

The scale internal consistency was assessed by obtaining the Cronbach’s alpha coefficient, which is indicated for Likert-like scales with a number of answers higher than two, and which evaluates whether the total variance of the test results is associated with the sum of the variance for each item^(^ 13 ^)^.

Interobserver reliability was determined by calculating the kappa coefficient^(^ 14 ^)^, and temporal stability was expressed by Pearson’s correlation coefficient. The interpretation of the magnitude of the kappa agreement estimators is, by convention: ≥ 0,75 (excellent); 0,40 a 0,75 (sufficient/good); e < 0 ,40 (weak)^(^ 14 ^)^.

Data statistical treatment was performed by using the SPSS version 23.0 software.

The present study was developed in the scope of the project *Gestão do Risco de Queda em Equipamentos para Idosos*, approved by the Ethics Commission at the Universidade Católica Portuguesa. The ethical principles in the Declaration of Helsinki were met in the execution of the present study, namely informed consent, privacy, and confidentiality^(^ 15 ^)^.

## Results

The sample was 102 elderly people with cognitive decline (MMSE ≤ 15 points for illiterate people, ≤ 22 for people with up to 11 years of formal education, and ≤ 27 for people with more than 11 years of formal education)^(^ 11 ^)^ of both genders, with 73.5% of women and 26.5% of men, institutionalized for 43.29 ± 41.64 months, and 69.6% at least 85 years old (Table 1).

**Table 1:** Characterization of elderly people who lived in two LTCIs regarding age, gender, fall occurrence, level of dependence, and use of auxiliary gait devices. Lisbon, Lx, Portugal, 2018

Sample characterization	
Age (years) ≥ 65 and < 75 ≥ 75 and < 85 ≥ 85	Percentage 2.9% 27.5% 69.6%
Gender Female Male	73.5% 26.5%
Fall episodes over the previous year Yes No	40.2% 59.8%
Recurrent fall Yes No	73.4% 26.6%
Injury resulting from the fall Yes No	51.3% 48.7%
Level of dependence None (independent) Low Intermediate High	18.8% 26.6% 49.2% 5.4%
Gait Does not use an auxiliary gait device Uses an auxiliary gait device Stick Crutches Walker Wheelchair (independently) Wheelchair (needs help)	8.9% 90.1% 4.0% 10.9% 10.9% 1.0% 61.4%

Although the present study did not have the objective of identifying fall prevalence, monitoring this indicator allowed to detect that it was high in both LTCIs. It was found that 40.2% of the elderly people experienced at least one fall and 73.4% fell two or more times over the past year.

Table 2 shows kappa agreement coefficient (k) values for each one of the items of the SPBIEPPF safety practices and behaviors dimension. It was not possible to determine the coefficient for items 7 and 8 because they showed constant values, that is, both observers chose the same answer option. For the other items, the values varied from 0.369 to 0.922. The indicators “opts to wear shoes with non-slip soles”, “organizes their bedroom’s space to facilitate moving in it”, “removes obstacles that hinder gait in the bedroom”, “ensures that their feet are well supported on the ground before standing”, and “verifies if the bathroom floor is not slippery/wet before using the room” showed an excellent level of agreement, statistically significant for p < 0.001.

**Table 2:** Determination of interobserver agreement as expressed by the kappa agreement coefficient. Lisbon, Lx, Portugal, 2018

Safety practices and behaviors dimension			
Indicator	κ*	p^†^	agreement
1. Seeks to persevere in choosing the best fall prevention measures	.789	< 0.001	Excellent
2. Selects shoes that are suitable for their feet	.369	< 0.001	Weak
3. Opts to wear closed-toe shoes	.712	< 0.001	Good
4. Opts to wear shoes with non-slip soles	.922	< 0.001	Excellent
5. Organizes their bedroom's space to facilitate moving in it	.882	< 0.001	Excellent
6. Removes obstacles that hinder gait in the bedroom	.826	< 0.001	Excellent
7. Keeps the bed wheels stopped	.000^‡^		
8. When getting up off the bed, first seats, with their feet well supported on the ground, and only then stands up	.000^‡^		
9. Ensures that their feet are well supported on the ground before standing	.841	< 0.001	Excellent
10. Verifies if the bathroom floor is not slippery/wet before using the room	.853	< 0.001	Excellent
11. Ensures that the floor is not slippery before carrying out hygiene care	.370	< 0.001	Weak

* κ = kappa agreement coefficient;

† p = significance probability value;

‡ .000 = no statistical parameter was calculated because the values were constant

The SPBIEPPF safety practices and behaviors dimension showed very good internal consistency, with a total Cronbach’s alpha equal to 0.895 for its 11 items, and had the evaluation performed by both Observer 1 (α = 0.843) and Observer 2 (α = 0.854).

Considering the obtained kappa values and the fact that there was a one-week interval between the two evaluations, the authors opted to calculate temporal stability by obtaining Pearson’s correlation coefficient. The calculated value (r = 0.936; p < 0.001) indicated that there was a very strong, positive, and very significant correlation between the two evaluations.

Regarding the evaluation of practices and behaviors of elderly people with cognitive decline, according to the data collected by observation and taking into account that the values associated with each one of the items ranged from 1 to 3, it was found that the examined institutionalized elderly people with cognitive decline had good fall prevention practices (Table 3).

**Table 3:** Sample characterization regarding practices and behaviors to prevent falls. Lisbon, Lx, Portugal, 2018

Indicator	Mean	Median	SD*
Practices and behaviors total result	31.45	33.00	2.34
1. Seeks to persevere in choosing the best fall prevention measures	2.83	3.00	.375
2. Selects shoes that are suitable for their feet	2.90	3.00	.299
3. Opts to wear closed-toe shoes	2.95	3.00	.217
4. Opts to wear shoes with non-slip soles	2.59	3.00	.569
5. Organizes their bedroom's space to facilitate moving in it	2.59	3.00	.569
6. Removes obstacles that hinder gait in the bedroom	2.91	3.00	.285
7. Keeps the bed wheels stopped	3.00	3.00	.000
8. When getting up off the bed, first seats, with their feet well supported on the ground, and only then stands up	3.00	3.00	.000
9. Ensures that their feet are well supported on the ground before standing	2.89	3.00	.312
10. Verifies if the bathroom floor is not slippery/wet before using the room	2.83	3.00	.375
11. Ensures that the floor is not slippery before carrying out hygiene care	2.95	3.00	.217

* SD = standard deviation

The totality of the safety practices and behaviors dimension (t = .842; p > 0.05) as well as the 11 items that make it up are not correlated with gender, but the totality of the dimension showed a correlation with institutionalization time (.355; p < 0.001). Analysis of the relationship between each one of the indicators and institutionalization time showed that the differences occurred in items 4 (rs = 0.220; p < 0.01), 5 (rs = 0.205; p < 0.01), and 10 (rs = 0.164; p < 0.05).

The residents who did not have gait alterations showed better practices (32.5 ± 31.2) when compared to those who had this type of alterations (29.00 ± 2.3), with the difference having a statistical meaning (t = 10.053; p < 0.001).

The elderly people who were 85 years old or older had better practices (31.7 ± 2.0) than those who were less than 85 years old (30.7 ± 2.9), with the difference showing a statistical meaning (t = -2.143; p < 0.05).

The possibility that fall prevalence was related to age was tested. However, the differences obtained did not have a statistical meaning (ϰ^2^ = .056; p > 0.05).

The association of fall occurrence in elderly people with cognitive decline over the previous year with their practices allowed to verify that the practices of the residents who fell during that period (31.7 ± 2.3) did not show a differences with a statistical meaning (t = 1.811; p > 0.05) when compared with those of the residents who did not fall (31.07 ± 2.3).

## Discussion

Falls, fall risk, and the fear of falling are focuses of attention for nurses’ clinical practice and are related to transitions in the health-disease process of elderly people and to the institutionalization process. This phenomenon is one of the reasons mentioned by relatives to seek an institution. Its repetition and consequences may lead to institutionalization, and it affects the residents who are still independent, given that they are living in a house designed for elderly people^(^ 16 ^)^.

The increase in the average life expectancy will be followed by a higher number of falls with injuries, which will interfere with institutionalized elderly people’s quality of life. For this reason, some authors consider that fall prevalence must be an item of quality evaluation in LTCIs^(^ 17 ^)^.

Additionally, there is the fact that, after the first fall episode, elderly people impose restrictions to their activities or find themselves restrained by other people^(^ 1 ^,^ 18 ^)^ for fear of a new occurrence, which promotes dependence.

Taking into account this context and the absence of an instrument that assessed practices and behaviors of institutionalized elderly people with cognitive decline to help prevent falls, the authors of the present study tested the SPBIEPPF psychometric properties in a sample with 102 elderly people with cognitive decline resorting to four observers.

Reliability analysis showed that the 11 items of the SPBIEPPF safety practices and behaviors dimension showed a very good reliability level and had internal consistency. These results expressed the scale potential to measure practices and behaviors of elderly people with cognitive decline to prevent fall episodes.

It is important to emphasize that the initial version of the instrument has a dimension addressing communication practices, which is a core element in any fall prevention program. Some authors advocate that improving the communication about preventive measures and health promotion between residents and professionals may ensure that appropriate and specific interventions are carried out to reduce fall incidence^(^ 6 ^,^ 19 ^)^, which is especially high in people with cognitive alterations and increases costs with health resources^(^ 9 ^)^. However, communication alterations induced by cognitive decline, encompassing confusion periods, changes in verbal and nonverbal language, and processing alterations, among others, do not allow to assess this dimension in the population with cognitive deficit.

A study that applied the SPBIEPPF to institutionalized elderly people who did not have cognitive decline concluded that there is a depreciation in the communication about falls in LTCIs, which may reinforce a depreciation of the frequency with which risk factors contribute to fall episodes and the assumption that this phenomenon is natural in this age group^(^ 6 ^)^.

The authors of this study mentioned that the worst possible total value/obtained value ratio occurred for the practices and behaviors of communication about risk factors between elderly people and institution professionals dimension. An average equal to 7.52 ± 3.24 out of a possible total value equal to 30^(^ 6 ^)^ was obtained. Future studies must characterize how communication about risk factors, about preventive measures, and about fall episodes affecting people with cognitive decline is carried out, which involves essentially the different professionals and the elderly people who do not have cognitive decline who witness falls of residents with cognitive impairment.

The safety practices and behaviors of elderly people dimension is made up of two parts, the first addressing safe practices and behaviors in self-care (items 1 to 7) and the second dealing with practices and behaviors related to the accessibility in the physical space (items 8 to 11). These parts equally showed a slight difference in the value of Cronbach’s alpha, which was α = 0.895, in comparison with the value calculated when the scale was validated, which was α = 0.814.

This value indicated good internal consistency. Future studies must investigate whether the fact that elderly people with cognitive decline concomitantly show physical impairments, for instance gait alterations that imply the use of auxiliary gait devices, increases the incidence of safety practices in self-care and impel them to have more caution when planning the organization of the physical space to improve accessibility in it.

The results of the study already mentioned pointed out that the practices and behaviors that occur most often, on average, are those related to self-care, namely choosing shoes with non-slip soles (4.06 ± 1.11) and close-ended shoes (3.45 ± 1.35) and getting up off the bed in a specific way, in which “most times” elderly people ensure that their feet are well supported on the ground before standing up (3.97 ± 1.27)^(^ 6 ^)^.

Regarding the evaluation of the interobserver agreement index, the present study found that seven out of the 11 items obtained good to excellent agreement. Based on these results, it is possible to consider that these items showed a good agreement among the experts and a good reproducibility concerning the assessment of practices and behaviors by hetero-observation.

Items 2 and 11 showed a low interobserver reliability. The difficulty in obtaining consensus may be a consequence of the nature of the activity, that is, the observers may have different assumptions about the capacity that elderly people with cognitive decline have to select proper shoes or ensure that the floor is not slippery.

Because of the importance these variables have in the mechanism of fall episodes and their prevention, they must be explored in future studies.

Items 7 and 8 showed constant values, which did not allow the determination of the kappa value.

It is possible that the results may have been influenced by random mistakes or measurement bias. One of the factors that may have affected them is the prejudice professionals may have against cognitive decline and against the skills a person in this situation may have.

The researchers that will apply this instrument in future studies must address this aspect with the observers. These have to be trained about the construct, its descriptors, the ideal conformity index, evaluation criteria, the standardization of the evaluation process^(^ 20 ^)^, and how differences and individual perceptions may bias objective evaluation.

Despite these difficulties, the findings pointed to the reliability of the indicators and of the instrument, indicating objectivity in the measurement. It is important to emphasize that reliability and agreement are not established properties of measurement instruments. Instead, they result from the interaction between the tool, the subjects or objects, and the context in which the evaluation is carried out^(^ 20 ^-^ 21 ^)^.

Statistical analysis showed that the practices and behaviors of elderly people with cognitive decline were good, given that the sample scored 31.45 out of a total of 33 points. The indicators with higher averages were “keeps the bed wheels stopped” and “when getting up off the bed, first seats, with their feet well supported on the ground, and only then stands up”. This practice may impact fall prevention, because 25.7% of falls in LTCIs occur when people are getting up off the bed and 37.1% during gait^(^ 1 ^)^.

The results of the present study emphasized the difference in elderly people’s practices according to these people’s gait skills. The residents who had no gait alterations showed better practices (32.5 ± 31.2) when compared with those who had alterations of this nature (29.00 ± 2.3). This factor may increase fall risks, because it adds to a major risk (gait alterations) unsafe practices related to self-care and accessibility in the physical space, both relevant risks in the genesis of the fall mechanism^(^ 1 ^,^ 3 ^-^ 5 ^)^.

There was no association between the occurrence of fall episodes over the previous year and the practices and behaviors of elderly people with cognitive decline. The instruments used to evaluate the fear of falling have not been validated for the population with cognitive decline, but this alteration contributes to determining the perception of fall occurrence and of the fear of falling.

The issue under discussion is complex and raises questions about the systematization and organization of nursing care. It is important that teams be trained^(^ 22 ^)^ and programs structured toward interventions that include training, leadership, mutual support, monitoring, and communication^(^ 23 ^)^.

Educational programs intended for professionals are a positive cost-effectiveness method to improve strategies oriented toward fall prevention^(^ 24 ^)^. The intervention must predict not only the approach to control biophysiological and environmental risk factors, but also practices and behaviors, especially of elderly people with cognitive decline, so professionals keep a closer look at them and help more fragile elderly people keep safe.

The limitations of the present study were related to the sample and the latent variable that was examined. Evaluating the practices and behaviors of elderly people with cognitive decline is a complex process, which requires training, experience, and time and, because doing it systematically in LTCIs is not habitual, the observers may have been influenced by their self-perception of the behavior of the residents with cognitive decline.

The absence of studies on practices and behaviors in the management of fall risk and the characteristics of the examined population hindered data discussion.

## Conclusion

The psychometric characteristics of the scale and the association of the items with preventive measures that help control the main fall risks indicated that the instrument under discussion is useful to evaluate the practices and behaviors of elderly people regarding fall prevention.

Internal consistency analysis showed that the scale had α = 0.895 for its 11 items, which indicated its capacity to evaluate the latent variable.

Regarding the indicators’ agreement and reliability, expressed by Cohen’s kappa coefficient, the results pointed out that the instrument has good reproducibility, which showed that it is valid and reliable to measure the practices and behaviors of institutionalized elderly people with cognitive decline to prevent falls.

It was verified that the practices and behaviors of elderly people with cognitive decline regarding the management of fall risks were not associated either with gender or the occurrence of a previous fall episode, but instead are influenced by institutionalization time, being older than 85 years, and gait skills.

Determining the SPBIEPPF psychometric properties allows it to be used with statistical relevance in research and in the nursing practice when applied to elderly people with cognitive decline who live in LTCIs.

Future studies must associate communication, safety practices and behaviors in self-care, and practices and behaviors related to accessibility in the physical space with fall risk, fall prevalence, fall mechanism, and the fear of falling.
